# On Evidence-Based Practice in Disaster Risk Reduction

**DOI:** 10.1007/s13753-021-00381-3

**Published:** 2021-11-15

**Authors:** David E. Alexander

**Affiliations:** Institute for Risk and Disaster Reduction, University College London, London, WC1E 6BT UK

**Keywords:** Case histories, Disaster risk reduction, Evidence-based practice, Policy formulation

## Abstract

Disaster science and scholarship are forever expanding and there are increasing calls to base disaster risk reduction policies on the evidence produced by such work. Using examples and argument, this opinion piece examines the nature of evidence. It defines evidence-based practice and considers how it has developed and become important to disaster risk reduction. A definition of what constitutes evidence is difficult to achieve but it must be made in relation to whether the data and information collected can usefully be interpreted and employed to change things for the better. Case histories from past and present centuries show that evidence can sometimes be argued over endlessly. In other cases it is roundly ignored. In yet other instances, false conclusions derived from evidence can become evidence in their own right. Nevertheless, there are situations in disaster risk reduction in which evidence is sorely needed but is clearly lacking. The effectiveness of counter-terrorism measures is one such area. In conclusion, evidence is valuable, above all if there is willingness to use it to support policy formulation, especially in a simple, transparent manner. Subjective interpretation can never be entirely removed from the use of evidence, and evidence alone will not stimulate the policy formulators to improve their decision making.

Now, what I want is, Facts. Teach these boys and girls nothing but Facts. Facts alone are wanted in life. Plant nothing else, and root out everything else. You can only form the minds of reasoning animals upon Facts: nothing else will ever be of any service to them. This is the principle on which I bring up my own children, and this is the principle on which I bring up these children. Stick to Facts, sir!—Thomas Gradgrind, in *Hard Times* by Charles Dickens (1854)

## Introduction

How much do governments, regulators, and risk managers want facts, and what do they consider to be evidence? At 0:54 a.m. on Wednesday, 14 June 2017, fire broke out in Grenfell Tower, a 24-story residential building in the Royal Borough of Kensington and Chelsea, London, England. The building burned for 24 hours, and at one point during the night a sheet of flame enveloped one entire side of the structure. Many people were trapped in their apartments. Seventy-two died, a similar number were injured and 400 people were left homeless (Moore-Bick 2019). In the subsequent official enquiry, the London Fire Brigade was severely criticized for adopting a stay-put-and-wait-to-be-rescued tactic when it had failed with fatal consequences in a previous apartment building fire in London, that of Lakanal House in 2009 (Moore-Bick 2019). Companies that had contributed to the recladding of Grenfell Tower with highly flammable panels were criticized for playing fast and loose with fire safety tests (Booth 2021). The tenancy management company was criticized for ignoring reasoned complaints about safety from residents of the tower block (Moffat 2017) and for failure of oversight regarding renovations to the building that transformed it into a potential towering inferno (Architects for Social Housing 2017).

Given modern knowledge of fire safety (Lane 2018), it should have been inconceivable that a fire tragedy of this sort would occur in the second decade of the twenty-first century. In previous decades, evidence from both the laboratory and the field had comprehensively defined the parameters of fire safety for high-rise buildings (Hayes 2017). Grenfell Tower was situated in one of the richest local authorities in the United Kingdom, so lack of resources could not be blamed. Evidence revealed that fire safety concerns had been routinely ignored or manipulated so that it did not threaten company profits (Hohmann 2019). The regulatory process had been progressively reduced so that it did not interfere with such liberties (Bell 2018). This example is therefore one that calls into question the meaning of evidence, not from the perspective of the ability to acquire it, but regarding whether or how it is used in relation to public safety.

The reason for describing this case is that it illustrates that evidence can either support or “get in the way of” risk management, depending on how, or whether, it is used. In the so-called age of disinformation and “fake news” (Balmas 2012), evidence is all too easy to dispense with (Rothkop 1999). Evidence of the risks associated with fire in Grenfell Tower and similar buildings was both present and easily accessible before the 2017 tragedy (House of Commons 1999). In an age dominated by the policies of neoliberal deregulation, one could argue that the evidence supported the public interest, but it was not employed because it did not support the political ideology (Fenton 2011).

Despite these issues, there is good reason to believe in “evidence-based practice.” Logic demands that we take experience into account and that we consider all relevant knowledge pertaining to a problem before we decide how to solve it. Without such an approach, policymakers risk blundering around in the dark, and their policies risk being, at best inefficient, and at worst downright injurious. However, there are two main problems with evidence-based practice. One concerns the nature of evidence and the other refers to the way in which it is, or is not, used.

This article offers opinions on and discussion of the issues associated with using evidence to support decision making for risk and disaster reduction. It enquires into the meaning of the terms “evidence” and “evidence-based practice.” It then considers the dangers associated with ignoring or failing to make use of evidence when it is useful and objective and holds the key to risk management.

## What is Evidence-Based Practice?

As a field, evidence-based practice appears to have been born out of the health sciences and services (Sackett et al. 1996; Kitson et al. 1998; APA Presidential Task Force on Evidence-Based Practice 2006; Melnyk and Fineout-Overholt 2011). It “...embraces various permutations including evidence-based practice, evidence-based nursing, evidence-based guidelines, evidence-based decision-making, evidence-based policy-making and evidence-informed patient choice, to name but a few” (Rycroft-Malone et al. 2004, p. 82). Clearly, if routine surgery ends in the death of the patient nine times out of ten, there is a need to find the evidence of why this happens and use the knowledge to prevent mortality the next time that kind of surgery is practiced. However, not all medical practitioners and theorists are convinced by this reasoning.

According to Fairbrother et al. (2015), evidence-based practice is balanced by, and apparently incompatible with, critical realism, an inductive method that “involves moving forward in time and studying group-and environment-related factors and changes as they happen, thus constructing meaning in real time, realistically, as it happens” (Fairbrother et al. 2015, p. 5). If this is true, then at the least the concept is not all-embracing. A more extreme viewpoint was stated by French (2002), who concluded that there is no real difference between the two approaches to the gathering and utilization of knowledge, and that “‘evidence-based practice’ is commonly a euphemism for information management, clinical judgement, professional practice development or managed care” (French 2002, p. 250). Thus, he suggested that it is merely a label for what goes on normally in clinical judgement.

Despite the cavils, rationalists, who have been frustrated to see policy in fields such as disaster risk reduction determined by prejudice, hearsay, misguided instinct, and corruption, have called repeatedly for policymakers to heed the scientists. The assumption is that the “model of natural science” (Harvey 1969) provides us with an objective, replicable, unbiased insight into the world, its processes, and its affairs. This can be used as a support for policy making on the basis of what is likely to bear fruit and what is not likely to work. Carabine (2015) provided a rationale for this process and a structure for it in terms of international policy on disaster reduction. Others have written cogently about the role of evidence in guiding disaster planning decisions (for example, Hoard et al. 2005; Auf der Heide 2006). Thus, evidence-based practice has become something of a mantra among those who seek to improve disaster risk reduction (Cutter et al. 2014). But what is evidence?

## What is Evidence?

It is axiomatic that policy and practice should be based on as complete knowledge of a problem as the evidence will allow (Gaillard and Mercer 2013). That is why policy formulators use academics and advisors—they have a wide-ranging knowledge of the problem in question, its connotations, and the evidence that, properly interpreted, can lead to a solution. Equally axiomatic, evidence is no good without interpretation—plus the ability to interpret it without misleading people.

However, for any problem in society, economy, and ecology that begs to be solved, there are at least nine important questions that may well lack adequate answers. They are as follows:

- What exactly is evidence?
- To what extent is evidence a surrogate for direct experience, or, alternatively, how much evidence should be derived from experience and how much from indirect sources?
- How should evidence be verified? Is it verifiable?
- Leading on from the previous question, does “evidence” merely consist of objective data, or does it include subjective experience?
- Evidence of what? To what should the evidence be attributed?
- What is evidence capable of proving or confirming?
- What is the connection between evidence and wisdom?
- Can we successfully do without evidence?
- How much evidence is enough before decisions can be made?

Clearly, the answers to these questions will differ from case to case. In general, “evidence” is factual information that is capable of contributing to the solution of the problem, which has been obtained by objective methods, and that paints an objective picture of the situation under examination. The evidence must be as complete as is needed in order to draw conclusions, formulate policy, and develop strategies to implement solutions. The evidence should be verified, or at least be verifiable, by independent means.

**Fig. 1:**
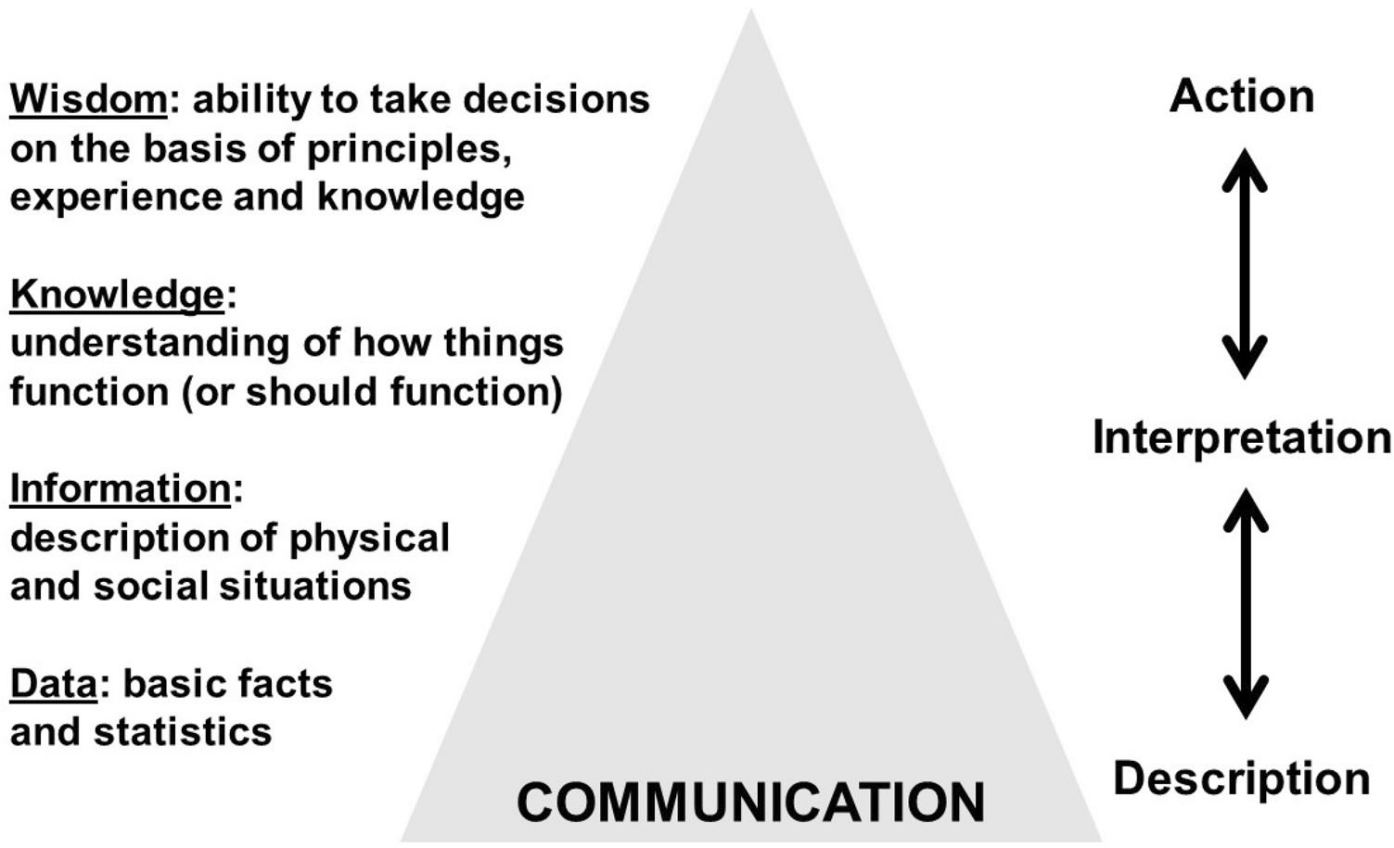
Modified data-information-knowledge-wisdom (DIKW) pyramid (after Henry 1974, and subsequent interpretations)

Merely trawling for data does not adequately define the process of compiling evidence. On the other hand, the inevitable retreat to selectivity risks the introduction of bias into the process of accumulating evidence. Moreover, as risk analysis involves risk perception, and as risk perception has a strong influence on how risks are communicated and managed, then subjective experience is clearly part of the “evidence” in some way. “Wisdom” is therefore the process of sifting and selecting evidence in an impartial and even-handed manner (Rowley 2007). In the modern world, information technology has promoted a massive return to the kinds of inductive science that were common in the times of the *Encyclopédistes* of the eighteenth century. Computers have taken the hard work out of blind analysis of data, but they have also removed the thinking. In intellectual terms, there is nothing more feeble and pathetic than data mining—unless, that is, it can be underpinned by a strong basis of knowledge and wisdom (Montgomery et al. 1989). One could argue that in recent times the so-called data-information-knowledge-wisdom (DIKW) pyramid has become progressively wider at the base and narrower at the top (Fig. 1).

## How Should Evidence be Used?

There are three kinds of evidence: precise and decisive; equivocal, ambiguous, and puzzling; and uninterpretable (evidence of what?). Data are a low-level form of evidence and may not be enough to form an adequate generalization about a phenomenon (Rowley 2007). Disaster impacts have two unfortunate features: over time, they are spiky and they have a (somewhat ambiguous) trend. Hence, it can be difficult to make a generalization about the future on the basis of evidence derived from the past. This was illustrated by an editorial in a journal, which congratulated the world on reducing disaster death tolls to 59,000 a year over the previous five years (Wilson 2005). It was published just as the Indian Ocean tsunami killed more people in one catastrophe than had died in all disasters during the previous 60 months. Unfortunately, for many phenomena, evidence alone will never be sufficient to characterize them, especially if their mean values trend over time, or there is not enough evidence to construct a robust magnitude-frequency relationship. Hence, if we need evidence, we also need models and inspiration. As all use of evidence is selective, the criteria by which facts are chosen should be made explicit so that they can be evaluated. In short, evidence can constrain uncertainty, but it cannot eradicate it. The next section describes a case in which the evidence could not be properly understood because the framework of interpretation did not exist and at the time could not be constructed.

## A Cautionary Tale

The Irish engineer Robert Mallet developed a strong interest in earthquakes. Indeed, he is to some extent the “Father of Observational Seismology.” One of his greatest achievements was to compile all the known evidence of earthquakes into a catalogue and world map of seismicity (Mallet and Mallet 1858). Mallet knew the location of plate boundaries before anyone knew of the existence of tectonic plates. In December 1857 the southern Italian region of Basilicata was struck by a major earthquake that killed about 5,000 people. Mallet organized an expedition there and assiduously collected evidence, often at great personal hardship, which he published in two volumes that have become classics of observational science (Mallet 1862). Mallet missed no piece of evidence, however trivial, but despite his impressive catalogue of the effects of seismicity, he was unable to deduce the cause of earthquakes, which he thought had something to do with subterranean steam. It took John Milne (1850–1913) to provide the missing interpretation, in concert with a number of other scientists, using a new and more sensitive kind of seismograph (Milne 1903).

## When Cautionary Tales Become Disaster: Post Hoc Evidence

**Fig. 2:**
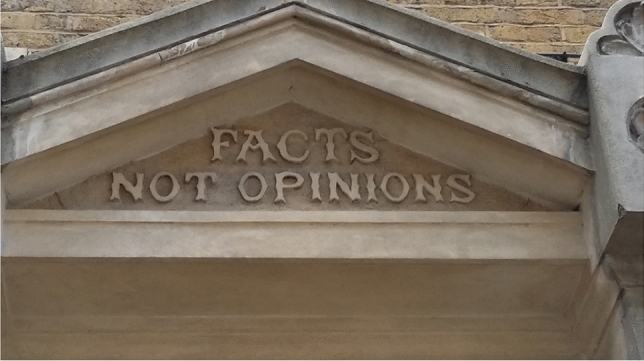
Entrance to the Kirkaldy Testing Company in London, now an industrial museum. Photograph by the author

The tympanum above the entrance door to the Kirkaldy Testing Company in Southwark Street, London, proclaims in chiselled stone “Facts, Not Opinions” (Fig. 2). David Kirkaldy (1820–1897) was a redoubtable Scottish engineer who in 1858 designed his own materials testing machine and seven years later installed it in his first London workshop. Among many other assignments, Kirkaldy’s establishment was involved in testing components of the Tay Bridge, which collapsed with a passenger train on it during a storm in December 1879. A Court of Inquiry was convened and much evidence was presented. No fewer than nine possible causes were debated, dealing with design, construction, workmanship, materials, maintenance, and oversight. Although the designer of the bridge, Sir Thomas Bouch (1822–1880), was held to be culpable, the evidence has been chewed over ever since, including by Kirkaldy, and rival etiologies have remained in circulation to the present day (Lewis 2004). This story is very reminiscent of the Vaiont landslide of 1963 in northern Italy, which killed 1,910 people and was also blamed (largely) on the designer of the Vaiont Dam, Ing. Carlo Semenza. Decades later, the evidence is still being reexamined and papers are still vigorously being published on the disaster, for example, Ibañez and Hatzor’s reevaluation (2018). The evidence is a magnet to researchers and each new generation of engineers sees something different in it. Meanwhile, major landslide and dam disasters continue to occur, but is that a testament to the inconclusiveness of evidence or simply failure to make proper use of it?

A more modern example illustrates a different point about the use of evidence. In 2015 a group of Western tourists climbed Mount Kinabalu in Malaysia and when they reached the top they took their clothes off. Photographs of this were widely circulated. Shortly afterwards, a magnitude 6.0 earthquake occurred, killing 18 people. Local wisdom had it that the god of the mountain was angry at the tourists for their lack of modesty. This viewpoint was publicly reiterated by the Deputy Chief Minister of the Malaysian State of Sabah, in which the mountain is situated (Pak 2015), although whether this was his sincere belief or merely a piece of populism cannot now be determined. On one level, the scientific one, this is a reminder of the kind of wrongful adduction of evidence that so preoccupied Sir James Frazer when he wrote that great paean to modernism, *The Golden Bough* (Frazer 1890). On the other hand, however wrong or ridiculous people’s views might seem, they *are* views and, because opinions condition actions, they too become part of the evidence.

## Let’s Ignore the Evidence

**Fig. 3:**
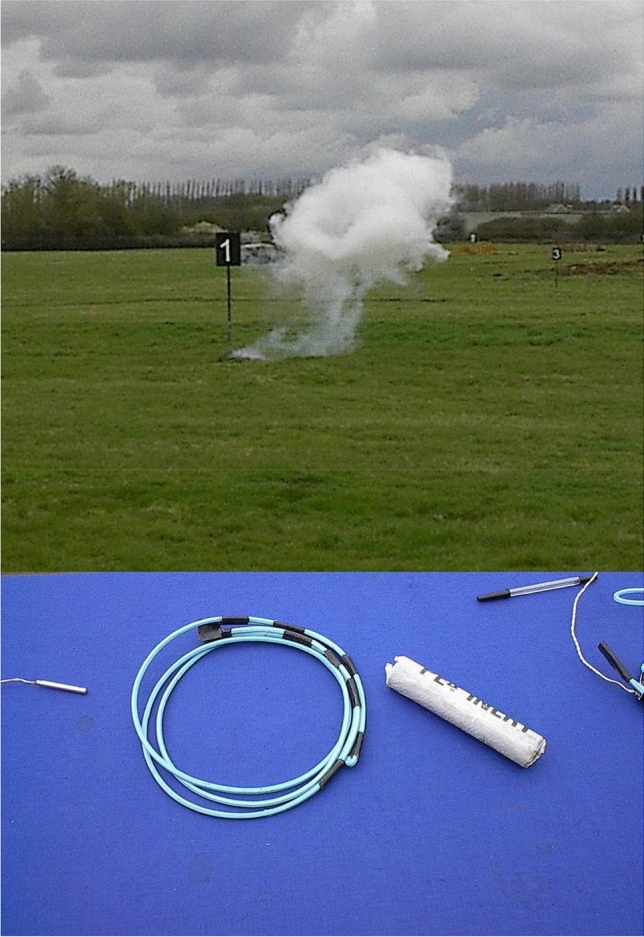
Plastic explosive and the end of a dummy terrorist. Photograph by the author

Another modern case is the following. Since 2006 there has been a set of international regulations that prohibit passengers from taking bottles of liquid larger than 100 ml onto civil aviation flights. This restriction stems from some assumptions about how liquids could be mixed on board an aircraft to make a bomb. To begin with, the standard sizes of bottles, at least in the European Union, are 60 and 120 ml. Hence, vast numbers of the latter have had to be thrown away at airports (the container size is what matters, not the amount of liquid inside the bottle). Secondly, in terms of concocting a bomb, 100 ml is definitely not a magic number. In a secure military environment, I asked a highly experienced counter-terrorism artificer about this and he told me that 25 ml of certain substances would be sufficient to make a viable and powerful bomb. I do not know whether one could buy the relevant substances in the airport pharmacy, having already passed security. Later on the same day I watched a dummy human being blown to tiny pieces by 150 grams of plastic explosive, an experience that was definitely food for thought (Fig. 3).

This example underlines the fact that there is seldom any attempt to evaluate the efficiency and effectiveness of counter-terrorism measures (Alexander 2011). The evidence is secret, elusive, or perhaps merely lacking. This state of affairs can easily lead to the suspicion that the evidence is inconvenient, especially for the military-political alliance and the security industry lobby.

On the one hand, counter-terrorism measures are perpetually growing more and more expensive. On the other, their effectiveness is seldom questioned. In 2006, the authors of a major desk-study on this concluded as follows:

After reading through the thousands of article abstracts from peer-reviewed sources, we also discovered that only 3.4% of them were based on studies that employed some type of empirical analysis on terrorism data or information. (Lum et al. 2006, pp. 491−492)

This situation did not improve significantly over the following decade.

## Let’s Ignore the Evidence When It Hits Us in the Face

A report from the United Nations International Strategy for Disaster Reduction (now the UNDRR) states that “The City of Venice joined the [Safe Cities] Campaign as a role model for cultural heritage protection and climate change adaptation” (UNISDR 2013, p. 1). Indeed, such an example is Venice that it appeared on the cover of this UNISDR document. In reality, Venice was severely threatened by the arrival of cruise ships, many of which are 11 storys high and weigh in at more than 90,000 tons. They navigated within a few meters of the historical urban fabric, causing damage with their bow waves and creating a massive risk of collision and shipwreck (Da Mosto et al. 2009), as well as polluting the atmosphere (Contini et al. 2011) and creating turbidity in the lagoon (Zaggia et al. 2015). Despite the example of the Costa Concordia (the world’s most expensive shipwreck (Alexander 2012)), in 35 years of debate the city council failed to legislate adequately on this issue (Casagrande 2015). Protests by Venetian residents eventually turned violent (Vianello 2017), but there was very little progress in tackling the hazard until UNESCO threatened to put Venice on its endangered list (Schemmer 2021). At that point, the Italian government issued a decree provisionally banning vessels of more than 25,000 deadweight tons from the Giudecca canal. No attempt was made adequately to regulate the chaotic smaller-scale water transport on the Grand Canal until an eminent German, Professor Joachim Vogel, was crushed and drowned in the collision between two boats (BBC News 2013). Meanwhile, the mayor of Venice, Sig. Giorgio Orsoni, resigned after being arrested in a corruption investigation regarding the city’s incomplete flood defences (Della Porta et al. 2015). So much for evidence-based practice—both within Venice and from outside looking in!

Here is another example of how evidence can be ignored. In 2016 the government of the United Kingdom made a policy that junior hospital doctors must work more hours for lower salaries at weekends. The rationale offered was that the quality of healthcare was lower at weekends and as a result more people were dying in hospitals on Saturdays and Sundays. In reality, a study (Meacock et al. 2017) showed that fewer people die in hospitals at weekends. Another study (Meacock et al. 2015) had already shown that a seven-day working week for doctors in hospitals would not be a cost-effective way of reducing mortality. The result of this was that the UK government pressed on with its policy, to the consternation of junior hospital doctors (McKay et al. 2016). Evidence could not be allowed to get in the way of a simple, straightforward diktat.

## The Use of Evidence in the Covid-19 Crisis

In the 30 months that elapsed after the Japanese tsunami of 11 March 2011, 2,600 papers and books were published on the Fukushima nuclear radiation release, its effects, and its aftermath (Povinec et al. 2021). This appeared to be something of a record for a single event. However, it was thoroughly eclipsed by the scientific effort on the Coronavirus tragedy. During the first three months of 2020, 6,600 papers on Covid-19 were published in the mainstream English-language scientific press. By mid-year the number had risen to 23,634 (Teixeira da Silva et al. 2021). At the end of 2020, *Nature* journal carried out an analysis of scientific publications on Covid-19 registered with the Scopus database and medRxiv preprint site and found that there were more than 100,000 articles (Else 2020). Papers on Coronavirus and its effects were appearing in print (or more likely in the digital equivalent) at a rate of one every three minutes. By December 2020 there were also at least 1,200 books on the disease in the English language alone. In other words, there was no shortage of evidence. Indeed, the challenge was to make sense of a quantity of evidence that was far, far too large for any human being to assimilate.

One parallel phenomenon was the abrupt creation of people who seemed suddenly to have acquired the ability to interpret disease data. As Clare Wenham (2020, p. 1335) wrote in *The Lancet*, “there has been an onslaught of armchair epidemiologists in the media.” I admit rather sheepishly to being one of them, as during 2020 I appeared on television and radio more than 70 times in discussions about the pandemic. At least I had been studying, teaching, and promoting pandemic emergency planning for the previous 12 years.

One of the most important questions during that pandemic has been the extent to which leading political decision makers have taken account of good scientific advice—in other words of the evidence as synthesized by those who fully understand it. Around the world, leadership has varied from intelligent to ignorant, humble to arrogant, sensitive to denialist, decisive to indecisive, engaged to negligent, supportive to exploitative, firm to erratic (Horton 2021). This signifies an extraordinary range of reactions to the evidence (Kaul et al. 2020). Moreover, there have been plenty of cases of failure to act upon imperatives highlighted by the results of scientific enquiry. At the same time, the science has included clear indications about the requirements of good leadership (Nicola et al. 2020).

To give the decision makers their due, the science of SARS-CoV-2 and the Covid-19 pandemic began from a point of relative ignorance. Factors that were not well understood at the start of 2020 included the disease’s infectiousness, reproduction number (R0), mutation rate, asymptomatic transmission potential, case-fatality rate, whether there would be several waves, its impact (by ethnicity, gender, and age-group), the acquisition of individual and “herd” immunity, how long immunity might last, the relationship of the disease to environmental factors, the efficacy of personal protective equipment, and what prospects existed for developing a functional vaccine against the disease. The response to this involved learning that was virtually unprecedented in its scope and rapidity. It also involved persistent controversy about almost all aspects of the disease and its actual or potential impact. None of this was helpful to people who were forced by circumstance to make operational decisions. Yet despite this uncertainty, in most cases, there was enough of a scientific consensus to assist them—but was the response fair and rational or merely politically polarized (Green et al. 2020)?

In 1971 Mr Zhou En-lai, the first Premier of the People’s Republic of China, was asked by Richard Nixon what he thought about the French Revolution of 1789. According to Nixon’s translator, he responded “it’s too early to tell” (Schama 1990, p. xv). Regarding Covid-19, it will take a long time for the evidence to be complete enough, to mature, in fact, for a clear verdict to be reached on how it has been used. Nevertheless, this does not mean that evidence is of marginal value. For example, in the wake of news that countries had used the Covid-19 pandemic as a pretext to curtail human rights or propagate abuses, the United Nations issued a report that talked about a “pandemic of human rights abuses” (UN 2020). Never has evidence been more sorely needed.

## Conclusion

William of Occam (1285–1347) stated the abductive heuristic *entia non sunt multiplicanda præter necessitatem*, literally “things should not be multiplied beyond what is required.” We can interpret this to mean that the explanation of a phenomenon should be the simplest one that is upheld by the evidence. A further implication is that more evidence does not necessarily mean better interpretations. With that in mind, evidence-based practice is a good idea providing we are not too naive about it. Any attempt to collect, marshal, and interpret evidence on a particular problem needs to be transparent, fair, and impartial. It must state the criteria by which evidence is included and excluded, and must ensure that an objective, balanced view of the problem is compiled. Besides the fact that they are grossly inefficient, inductive and adductive processes will not automatically ensure this. A “blind” approach to evidence will not make it objective or comprehensive, because choices inevitably have to be made in the way that evidence is collected.

Lastly, examples described in this commentary illustrate the fact that evidence alone does not “shame” policymakers into adopting a better, more objective approach. They are perfectly at liberty to use evidence selectively, or ignore it altogether.

Hence, we need an evidence-based investigation of exactly how and why policymakers ignore or manipulate the evidence.
